# A Simple All-Optical Water Level Monitoring System Based on Wavelength Division Multiplexing with an Arrayed Waveguide Grating

**DOI:** 10.3390/s19143095

**Published:** 2019-07-13

**Authors:** Hoon-Keun Lee, Jaeyul Choo, Gangsig Shin

**Affiliations:** 1 Department of Nuclear Safety Research, Korea Institute of Nuclear Safety, 62 Gwahak-ro, Yuseong-gu, Daejeon 34142, Korea; 2 Department of Instrumentation, Control and Electrical System, Korea Institute of Nuclear Safety, 62 Gwahak-ro, Yuseong-gu, Daejeon 34142, Korea

**Keywords:** optical fiber sensor (OFS), water level monitoring, wavelength division multiplexing (WDM), spent fuel pool (SFP)

## Abstract

We propose and demonstrate a simple water level monitoring system based on the wavelength division multiplexing (WDM) for the spent fuel pool (SFP) at a nuclear power plant. The basic principle is based on the measurement of the optical power spectra by the Fresnel reflection according to the change of the refractive index at the end facet of the optical fiber tip (OFT). An arrayed waveguide grating (AWG) is employed to achieve multi-channel sensing capability with a C-band broadband light source (BLS) based on amplified spontaneous emission (ASE). The feasibility of the proposed scheme is investigated with a simulation and experimentation. We also investigate the limiting factor for remote transmission. The system performance is degraded by the Rayleigh backscattering of the BLS light, but it can be operated over long distances within 10 km with 5 dB of difference peak power margin.

## 1. Introduction

The spent fuel pools (SFPs) of nuclear power plants are operated by plant owners within the specified operational limits and conditions (such as water level, temperature and radiation dose) for nuclear safety [1]. The water inside of the SFP plays an important role for the spent fuels in cooling down of the decay heat and shielding of the radiation fields [2]. During the Fukushima Daiichi accident, the plants lost their ability to cool the SFPs and monitor the level of water because of the loss of power resulting from the tsunami. This caused a failure to make a best decision among the priority tasks for the emergency responses within the restricted resources [3]. After this accident, the Nuclear Regulatory Commission (NRC) issued an order to require that all U.S. nuclear power plants install water level instrumentation in their SFPs with three distinct water levels that could be reported remotely [4]. From these reinforced regulatory circumstances, it is required to develop a new concept of remote or portable sensors that can monitor the water level continuously to enhance nuclear safety. 

Generally, the water level in the SFP is measured with an ultrasonic level meter. This level meter provides the advantages of simplicity and continuous level measurements. However, there are some limitations, due to its limited measurable distance and low resistance to electromagnetic interference (EMI). Moreover, it is not easy to operate the level meter during a loss of power accident. To overcome these limitations, there have been several approaches to apply an optical fiber sensor (OFS) to nuclear facilities, due to its advantages such as passive sensing capability, remote transmission, EMI-tolerance, radiation-resistance and redundancy [5,6,7,8]. Specifically, many studies have been done with the various types of optical sensing techniques to monitor important physical parameters under harsh environments such as neutron and gamma radiation [8,9,10,11,12]. For the water level measurement, there have been reported a number of quasi-distributed optical fiber sensors based on several multiplexed sensing techniques [6,7,13,14,15,16,17,18]. The multiplexing schemes are divided into two categories according to the sensor types: (1) discrete level sensors with an optical coupler, or (2) special fiber sensors that embedded several discrete level sensors on a single fiber. One of the simple multiplexing methods is utilizing the time division multiplexing (TDM) technique based an optical time domain reflectometer (OTDR) with an optical coupler [6,7,13]. It can be implemented easily with commercially available optical components, but this method suffers from the high insertion loss of the coupler to enhance the spatial resolution of the water level. Another simple method is employing a specially fabricated single fiber sensor with multiple discrete level sensing functions. For this purpose, several options have been proposed, such as arrayed fiber Bragg grating (FBG) sensors embedded in silicone rubber [14], optically heated FBG arrays [15], a plastic optical fiber with laterally polished bent sections [16] or engraved grooves [17], a single mode fiber with uniformly spaced multiple gaps [18], and so on. However, this method still suffers from the limited measurement points resulting from the fabrication process. In addition, some of the proposed sensors require previously measured reference information such as wavelength [14,15], or received power [16,17], or signal processing for cross-correlation [18]. 

In this paper, for the first time to our knowledge, we propose and demonstrate a very simple all-optical water level monitoring system based on wavelength division multiplexing (WDM) with an arrayed waveguide grating (AWG). The AWG plays a role of multiplexer and de-multiplexer at the same time for the measurement of the reflected optical signals [19]. The proposed method can provide a multi-channel sensing capability (more than 32 channels with 100 GHZ channel spacing) effectively at remote and onsite locations with a low insertion loss. Moreover, it does not need previously measured information (i.e., the reference values) for comparison with the currently updated status or any signal processing technique for the received reflection signal. Thus, it is possible to provide a fast response for level measurement in an emergency situation for a nuclear power plant. Above all, the proposed scheme can be implemented easily with commercially available optical components and devices. In this experiment, a C-band (1530–1565 nm) broadband light source (BLS) based on amplified spontaneous emission (ASE) was employed to check the feasibility of the proposed scheme with 11 AWG channels. We verified the feasibility of the proposed method with a simulation, and the limiting factor is also investigated for remote transmission.

## 2. Architecture of the Water Level Monitoring System

**Figure 1:**
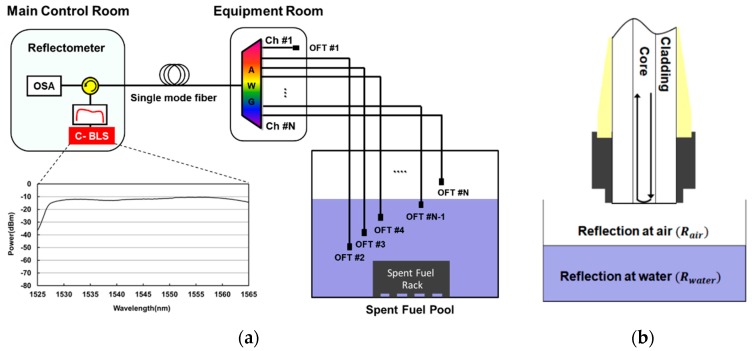
(**a**) Configuration of the proposed all-optical water level monitoring system, and (**b**) schematic drawing of optical fiber tip.

The architecture of the proposed water level monitoring system is shown in Figure 1a. It consists of a reflectometer for optical power measurement, a 1×N AWG for channel multiplexing/de-multiplexing and optical fiber tips (OFTs) for the sensing the water presence. A simplified structure of the OFT is represented at Figure 1b. It is based on a single mode fiber including the standard SC/PC type connectors with a ceramic ferrule diameter of 2.5 mm at the ends of the fiber. Then, the reflectometer is made up of a BLS based on ASE for optical source, an optical circulator and an optical spectrum analyzer (OSA). The inset of Figure 1a shows the measured optical spectrum of the BLS with the resolution bandwidth of 0.2 nm. The ASE light for BLS is generated by a pumped erbium-doped fiber. It can provide a large bandwidth (more than 30 nm) with the gain flattened output spectrum (2.5 dB power deviation at maximum).

For the monitoring of the WDM channels at the remote location, the reflectometer is installed in the main control room. The optical signal path for the reflectometer is as follows: First, the ASE light from the C-band BLS is transmitted to the AWG after passing through the optical circulator and a few km of single mode fiber. Then, the ASE is spectrum-sliced by the AWG in the equipment room and each spectrum-sliced ASE light is sent to OFTs connected with the dedicated wavelength channels of the AWG. The AWG are installed in a loop controller cabinet in the equipment room with environmental management such as temperature, humidity and dust, etc. Then, the spectrum-sliced ASE is reflected at the end facet of the fiber tip according to the refractive index of materials (air or water). Finally, the reflected spectrum-sliced ASE lights are detected by the OSA in the reflectometer after re-passing the AWG and the optical circulator via the single mode fiber. As a result, the water level is monitored continuously at the remote location. Two channels of the AWG (channel #1 for primary and #N for back-up) are used as the reference channels to identity the presence of the water by comparing with the reflected optical signals between another sensing channels and the reference channels. The reference channels also helps to effectively recognize the start and end wavelength point within the designed wavelength region.

It may be noted that the reflectometer also could be employed for the purpose of hand-held monitoring when a severe accident happens, such as a station blackout at a nuclear power plant. This is because the reflectometer could be miniaturized with a low cost and simple configuration by substituting the BLS based ASE with a reflective semi-conductor optical amplifier or a Fabry-Perot laser diode [19]. They have a large spectral bandwidth with a low front facet reflectivity for WDM applications.

## 3. Simulation and Experimental Results

### 3.1. Operation Principle 

The ASE light source can provide a large bandwidth with the gain flattened output spectrum as shown in Figure 1a. Thus, the electrical field of BLS based on ASE light in the reflectometer can be simply modeled by the following equation [20],

(1) $$E_{ASE}(\lambda )=\left|E_{ASE}\left(\lambda \right)\right|e^{i\varphi }$$

where $\left|E_{ASE}\left(\lambda \right)\right|$ is a constant amplitude in wavelength domain (λ) and $e^{i\varphi }$ represents a uniformly random distributed phase within the range of 0–2 π. The field of the spectrum-sliced ASE light $\left(E_{SSA}\left(\lambda \right)\right)$ can be obtained by multiplying the square root of the AWG transfer function with the generated ASE ($E_{ASE}\left(\lambda \right)$).

(2) $$E_{SSA}(\lambda )=\sqrt{T_{AWG}\left(\lambda \right)}\cdot E_{ASE}\left(\lambda \right)$$

Here the ideal transfer function of the AWG can be given by:

(3) $$T_{AWG}\left(\lambda \right)=exp\left[-ln2{\left(\frac{\lambda -\lambda_{c}}{\Delta \lambda_{BW}/2}\right)}^{2m}\right]$$

where m is the filter-order for super-Gaussian shape, $\lambda_{c}$ and $\Delta \lambda_{BW}$ represent the center wavelength and the 3 dB bandwidth of the AWG, respectively. The values of parameters used for the modeling of the AWG are as follows: m = 1.415, $\lambda_{c}$ = 1550 nm and $\Delta \lambda_{BW}$ = 0.64 nm. After passing through the AWG, the field of the spectrum-sliced ASE arrives at the end facet of the OFT. The principle of level sensing is based on the Fresnel reflection occurring at the fiber tip according to the change of refractive index [21]. The Fresnel power reflection coefficients of air ($R_{air}$) and water ($R_{water}$) at the fiber tip can be given by:

(4) $$R_{air}={\left(\frac{n_{f}-n_{a}}{n_{f}+n_{a}}\right)}^{2},R_{water}={\left(\frac{n_{f}-n_{w}}{n_{f}+n_{w}}\right)}^{2}$$

with $n_{f}$ being the refractive index of the fiber, $n_{a}$ and $n_{w}$ being the refractive indices of the air and water, respectively. The value of $n_{a}$ is 1.0002739 [22]. The parameter values of $n_{f}$ and $n_{w}$ can be obtained by using the Sellmeier formula [22,23,24], and it can be approximated as a constant within C-band. In this simulation, we assumed $n_{f}$ of 1.4492 and $n_{w}$ of 1.3154 at 1550 nm.

Based on the estimated refractive indices, the theoretical values of the Fresnel reflection coefficient are about −26.3 dB for the water ($R_{water}$) and −14.7 dB for the air ($R_{air}$), respectively. The reflected (peak) power difference (ΔP) between at the air and water is about 11.6 dB. Generally, the refractive indices of materials are not only dependent on wavelength, but also temperature [25]. Unfortunately, the temperature of the water in the SFP can be changed by the external environmental condition such as the trips of recirculation cooling pumps. For the water, the thermo-optic coefficient (temperature dependence on the refractive index) is about $-8\times 10^{-5}$/°C [26,27,28] and the temperature behavior of the refractive index can be approximated with a linear expression [29]. In calculation, the variation of the reflected power at the water is less than 0.35 dB within the external temperature of 20–80 °C. In spite of the power variation according to the temperature, ΔP can be still maintained at more than 11 dB. In contrast with the water, the thermo-optic coefficient for the optical fiber is about $8.6\times 10^{-6}$/°C [28,29,30], meaning the effect of the temperature variation can be negligible for the optical fiber [30].

Then, the reflected spectrum-sliced ASE field ($E_{R-SSA}\left(\lambda \right)$) at the fiber tip can be represented by multiplying the square root of the Fresnel reflection coefficient as below.

(5) $$E_{R-SSA}^{air}\left(\lambda \right)=\sqrt{R_{air}}\cdot E_{SSA}\left(\lambda \right),\;E_{R-SSA}^{water}\left(\lambda \right)=\sqrt{R_{water}}\cdot E_{SSA}\left(\lambda \right)$$

By combining the Equations (1)–(5), the intensity of the received optical spectrum ($I_{output}\left(\lambda \right)$) at the OSA in the reflectometer can be expressed as Equation (6) below. It may be noted that the BLS is passing the AWG twice for multiplexing and de-multiplexing.

(6) $$I_{output}\left(\lambda \right)=10^{\left(\frac{-L_{IL}}{10}\right)}\cdot R_{m}\cdot {\left|E_{ASE}\left(\lambda \right)\cdot T_{AWG}\left(\lambda \right)\right|}^{2}$$

Here, $L_{IL}$ is the total insertion losses of the signal reflection path in dB scale and $R_{m}$ is the Fresnel reflection coefficient according to the materials (air or water). The total insertion losses can be expressed with Equation (7) below, by adding all of the insertion losses of each optical component.

(7) $$L_{IL}\left[dB\right]=2L_{AWG}+2L_{OFT}+L_{OC}+2L_{Fiber}$$

In this simulation, we estimated total insertion losses ($L_{IL}$) of 12.5 dB, including the AWG ($L_{AWG}$: 4.5 dB), the OFT ($L_{OFT}$: 1 dB) and the optical circulator ($L_{OC}$: 1.5 dB) at the back-to-back condition (i.e., without considering the transmission length). These insertion losses are based on the measurement result.

### 3.2. Experimental Setup and Results

To simplify the experimental setup, we utilized only the 10 nm wavelength band (1545–1555 nm) of the BLS with the help of the optical band-pass filter. It can accommodate 11 channels of the AWG at 100 GHz channel spacing. A variable optical attenuator was positioned between the BLS and the optical band-pass filter to adjust the transmission power to the AWG. The measured BLS power was about −11.2 dBm/0.2 nm (total power: +6 dBm) after passing the optical circulator. The BLS output was spectrum-sliced by the flat-top passband AWG. The channel spacing and its 3 dB bandwidth were 100 GHz (0.8 nm) and 80 GHz (0.64 nm), respectively. The measured spectrum-sliced ASE power was about −15.7 dBm/0.2 nm (total power: −10.5 dBm) at the selected channel #6 (center wavelength: 1550 nm). The OFTs were connected with the 11 channels of the AWG, and their lengths and losses of the OFTs were about 2 m and within 1 dB, respectively. The tips were immersed in the water tank with the height of 30 cm at room temperature. 

**Figure 2:**
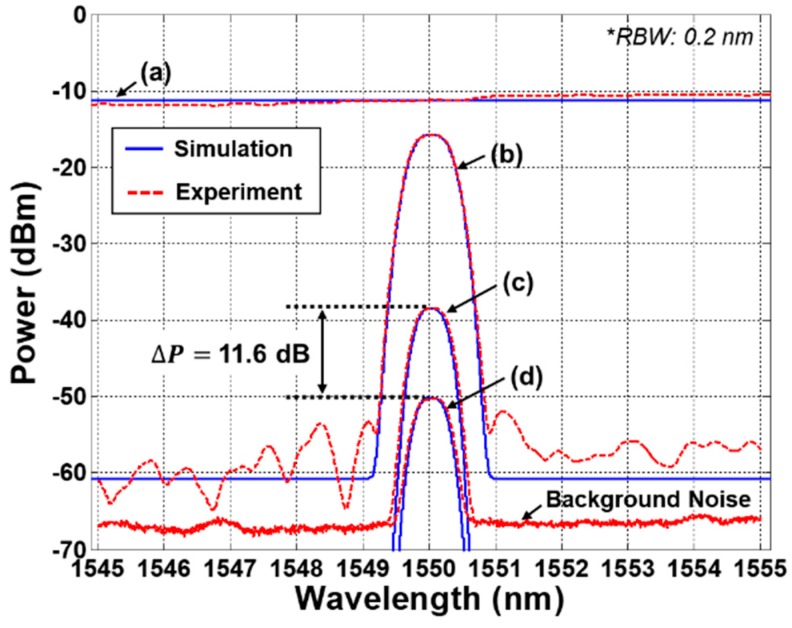
Simulated and measured optical spectra of the spectrum-sliced amplified spontaneous emission (ASE) according to the Fresnel reflection. (**a**) ASE, (**b**) spectrum-sliced ASE, (**c**) spectrum-sliced ASE reflected at the air, and (**d**) spectrum-sliced ASE reflected at the water.

The measured optical spectra of channel #6 were shown in Figure 2 with the simulation results. The optical spectra of (a) and (b) represent the ASE lights before and after spectrum-slicing by the AWG. The insertion loss of the AWG was 4.5 dB. The spectra of (c) and (d) show the received ASE lights after reflection at the air and the water, respectively. The measured peak power of the spectrum-sliced ASE reflected at the air and the water was about −38.5 dBm/0.2 nm and −50.1 dBm/0.2 nm, respectively. The peak power difference between two optical spectra was about 11.6 dB. These experimental results are well matched with the simulation results. It should be noted that the rest of the AWG channels were terminated with optical terminators to prevent the reflection of the unwanted ASE lights except the channel #6. As shown in Figure 2, the measured background power due to the reflection of the optical connectors was about −67.5 dBm/0.2 nm (including the 1.5 dB insertion loss of the optical circulator), which acted as background noise [31,32,33]. 

**Figure 3:**
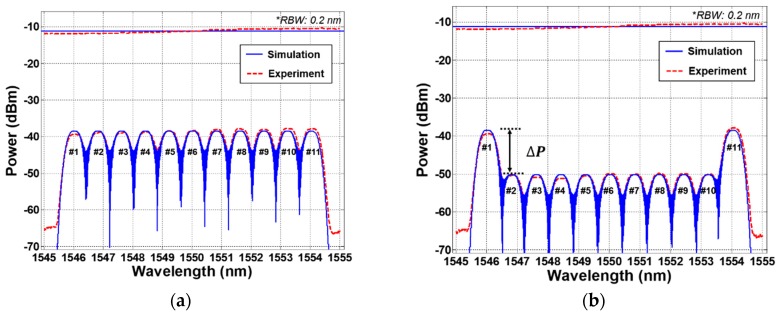
Simulated and measured optical spectra of the reflected spectrum-sliced amplified spontaneous emission (ASE) with wavelength division multiplexing (WDM) channels (**a**) the lowest water level, and (**b**) the full water level.

To investigate the multi-channel sensing capability of the proposed scheme, we measured the spectra of the 11 channels of water level monitoring system. Figure 3a,b show the measured WDM optical spectra with the reflectometer at the lowest water level and at the full water level, respectively. The experimental results shows a good agreement with the simulation result. The first channel #1 and the last channel #11 are utilized as the reference power channels for primary and back-up, respectively, which means that the two reference channels are only measuring the peak power of the spectrum-sliced ASE reflected at the air. They can provide the reference information to identify the presence of the water for the remaining 9 sensing channels (channels #2–#10). In addition, these reference channels facilitate the recognition of the designed wavelength regions for the water level monitoring system. In this demonstration, the 10 steps of the water level can be presented with the 9 sensing channels from the lowest level of 0 step (1/111111111/1) to the highest level of 9 step (1/000000000/1) as shown in Figure 3. Here, ‘1’ and ‘0’ levels mean the presence of air and water, respectively. Thus, by measuring the peak power difference (ΔP) between the reference channels (#1, #11) and the sensing channels (#2–#10), the current status of the water level is monitored in real time. The peak power differences among the 9 sensing channels are less than 1 dB. These differences maybe result from various factors such as the different reflection path loss (including the AWG, OFT) and the spectral profile of the BLS output and so on. 

In this experiment, we demonstrated the water level monitoring system at the back-to-back condition for simplicity. However, the system performance could be degraded by the Rayleigh backscattering light of the BLS by passing through a long distance single mode fiber for remote transmission [31,32,33]. Assuming optical fiber loss of 0.25 dB/km, we calculated the Rayleigh backscattering power according to the transmission length. By increasing the transmission length, the background noise power increases due to Rayleigh backscattering. In spite of the fiber attenuation loss, the Rayleigh backscattering light resulting from the transmitted light source caused the increase of total received power at the reflectometer, and this effect introduced degradation of the system performance (i.e., decrease of the ΔP). To guarantee system performance regardless of the internal and external environment conditions, it is required to set up the minimum difference peak power margin for water level detection. If we consider the various degradation factors such as the BLS output spectrum deviation (2.5 dB), the temperature variation due to the external environment (0.5 dB) and the insertion loss deviation among channels for AWG and OFT (1 dB), the minimum difference peak power margin of 5 dB needs to be maintained including an additional power margin (1 dB). Based on this requirement, the allowable transmission length is about 10 km. It should be noted that the transmission length can be increased further with low-loss passive optical components. 

## 4. Discussion

By employing the WDM technique (a BLS and AWG) instead of the TDM (an OTDR and optical coupler), we can increase the spatial resolution of the measurement of the water level. For the case of the TDM, since the insertion loss of the optical coupler increases as the number of channels increases, there is a limitation to improve the spatial resolution. Moreover, it requires well designed optical fiber lengths according the channels to detect the reflected light with a delayed time. However, for the case of the proposed WDM scheme, the channel capacity can be increased easily by utilizing another wavelength band of BLS with the cyclic characteristic of the AWG [34] or reducing the channel bandwidth of the AWG [35]. Furthermore, it is possible to accommodate the same length of fiber cable for the OFT. Thus, it can provide reduced costs and increased efficiency of maintenance. 

In spite of these merits, the contact type of OFTs can be affected by the radiation induced attenuation resulting from the spent fuels in the pool. For the conventional single mode fiber (SMF28), the radiation induced attenuation was reported to be about 74 dB/km at 1550 nm with the total accumulated dose level of 1 MGy [36]. This accumulated dose level corresponds to the total amount of radiation that occurs over a period of 40 years in SFP [8]. However, this attenuation value is acceptable, because the OFTs actually immersed in the spent fuel pool will be less than 10 m long (radiation induced attenuation loss: ~0.74 dB/10 m) [6,8]. Moreover, this radiation induced attenuation level can be mitigated by employing OFTs based on radiation hardened pure-silica core or fluorine-doped optical fiber [8,11,12]. Another issue related to OFTs is the floating particles of dust or small particles dissolved in the spent fuel pool. These particles can also cause performance degradation by adhering to the surfaces of tips. However, this issue can be mitigated by the plant operators. The operators should manage the cleanliness and transparency of the water to an acceptable level in the SFP to protect the related facilities and to prevent corrosion of the cladding of the spent fuels according to the technical specification. This purification is carried out through one or two flow paths consisting of floating removal filters and ion exchangers. Maintenance activities such as periodic testing of the system performance will be helpful to monitor the condition of the OFTs. 

## 5. Conclusions

We have proposed and demonstrated a simple all-optical water level monitoring system based on the wavelength division multiplexing method for spent fuel pools in nuclear power plants. The feasibility of this system was investigated by simulation and experiment. This proposed system could provide a multi-channel sensing capability at a remote or onsite location. A 32 channel arrayed waveguide grating (channel spacing: 0.8 nm) was employed to achieve multi-channel sensing capability with a C-band (1530–1565 nm) broadband light source based on ASE. By monitoring the peak power difference between the reference channels and the sensing channels of the AWG, a real time measurement for the water level could be achieved. We also investigated the limiting factor for remote transmission with the simulation. The dominant noise source is the Rayleigh backscattering of the transmitted broadband light source, which makes the system performance degrade as the transmission length is increased. However, this system can be operated over long distances within 10 km with 5 dB of difference peak power margin. Based on the results of this research, it is expected that the proposed water level monitoring system can be used as an auxiliary monitoring system for a spent fuel pool. Further research will be needed to enhance the resistance of the optical fiber tips to the harsh environments of the spent fuel pool.
